# Acute Symptomatic Hyponatremia With Reversible Diffuse Brain Edema on CT

**DOI:** 10.7759/cureus.97960

**Published:** 2025-11-27

**Authors:** Yasutaka Yoshiike, So Sakamoto, Chikao Ito, Isao Takahashi

**Affiliations:** 1 Emergency Medicine, Asahi General Hospital, Asahi, JPN

**Keywords:** acute hyponatremia, brain edema, hypertonic saline, water intoxication, water restriction

## Abstract

Hyponatremia-induced altered consciousness is a common clinical presentation, and brain edema is a well-known complication. However, few reports describe detailed imaging findings or discuss the neurological prognosis in such cases. We report a case of acute symptomatic hyponatremia in which diffuse brain edema was evident on CT at admission. The imaging findings resolved with appropriate treatment during hospitalization, and the patient recovered with no neurological sequelae. This case demonstrates that brain edema caused by hyponatremia can be reversible and does not necessarily predict a poor neurological outcome. It also highlights the importance of considering water intoxication in patients presenting with hyponatremia and diffuse brain edema. Additionally, it suggests that CT findings may assist in guiding treatment strategies for severe hyponatremia.

## Introduction

Hyponatremia primarily causes central nervous system dysfunction. Symptoms range from mild ones such as nausea, vomiting, confusion, and headache to severe ones, including impaired consciousness and seizures. Acute hyponatremia tends to present with more severe symptoms, and we often experience unconsciousness and seizures. In cases of acute onset, brain edema may be visible on imaging studies; however, few reports detail such CT findings, and limited literature addresses the associated neurological prognosis.

We present a case of acute symptomatic hyponatremia in diffuse brain edema evident on CT at admission. Imaging abnormalities resolved during the hospitalization course with appropriate treatment, and the patient experienced no lasting neurological deficits.

## Case presentation

A 51-year-old man with a history of schizophrenia, residing in a care facility, presented with impaired consciousness. The facility staff did not know when he had become unconscious. He had been hospitalized multiple times in the past due to water intoxication, but we could not access past data such as blood laboratory data. His medications included olanzapine 2.5 mg/day, valproic acid 200 mg/day, flunitrazepam 2 mg/day, clonazepam 1.5 mg/day, and risperidone 9 mg/day. Facility staff reported witnessing the patient drink large amounts of water the day before admission. He was found by facility staff experiencing unconsciousness and convulsions and was transported to our hospital.

He was transported 90 minutes after discovery. During transport, the convulsions persisted. On arrival, vital signs were: temperature 35.8℃, blood pressure 151/107 mmHg, heart rate 89/min, respiratory rate 19/min, and SpO_2_ 100% (O_2_ 10 L). His Glasgow Coma Scale (GCS) score was 3. We administered diazepam 10 mg and levetiracetam 1,000 mg, but his convulsions did not stop. We ultimately initiated continuous IV infusion of midazolam, and the convulsions ceased.

We present the blood and urine laboratory data at admission in Table 1.

**Table 1:** Blood and urine laboratory data on admission

Laboratory Parameter	Result	Reference Range
Sodium (mEq/L)	105	138-145
Chloride (mEq/L)	73	101-108
Urea nitrogen (mg/dL)	5	8-20
Creatinine (mg/dL)	0.47	0.65-1.07
Creatine kinase (U/L)	845	59-248
Serum osmorality (mOsm/L)	219.8	275-290
pH	7.335	7.350-7.450
pCO_2 _(mmHg)	42.8	35.0-45.0
HCO3^- ^(mmHg)	22.3	20.0-26.0
Lactate (mmol/L)	3.1	0.5-1.6
Urine osmorality (mOsm/kg)	276	50-1300
Urine sodium (mEq/L)	51	110-250

**Figure 1:**
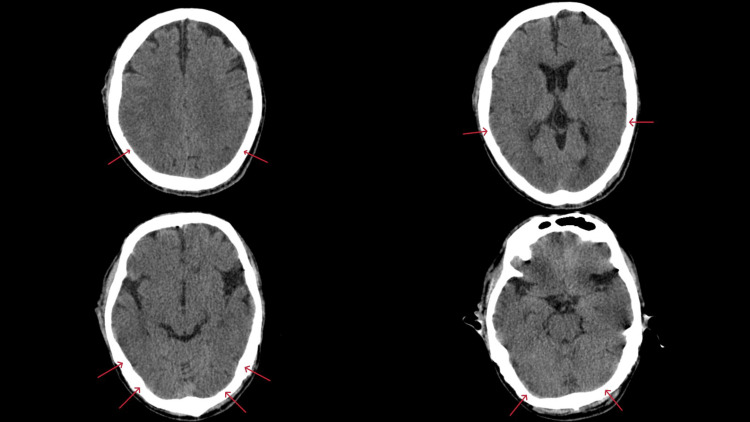
CT scan images on arrival show diffuse brain edema The sulci are markedly absent (red arrows), suggesting diffuse brain edema.

Head CT revealed diffuse brain edema with loss of brain sulci (Figure 1). Based on the history of excessive water intake, a diagnosis of acute hyponatremia was made due to water intoxication and accompanying diffuse brain edema. We initiated treatment with hypertonic saline (10% NaCl 60 mL + 500 mL normal saline, approximately 1.9% NaCl) in the emergency department. We intubated him due to unconsciousness and admitted him to the intensive care unit (ICU).

Five hours after admission, serum sodium had increased to 111.9 mEq/L, and we determined this represented overcorrection. Thus, we switched to administering normal saline. However, serum sodium levels continued to be overcorrected, rising to 117.3 mEq/L by eight hours after admission. Urine output ranged from 400 to 900 ml/hr. Therefore, we added desmopressin nasal spray (5 mcg). By Hour 24, serum sodium had risen to 121.5 mEq/L, which is an increase of 16.7mEq/L/24 hours.

**Figure 2:**
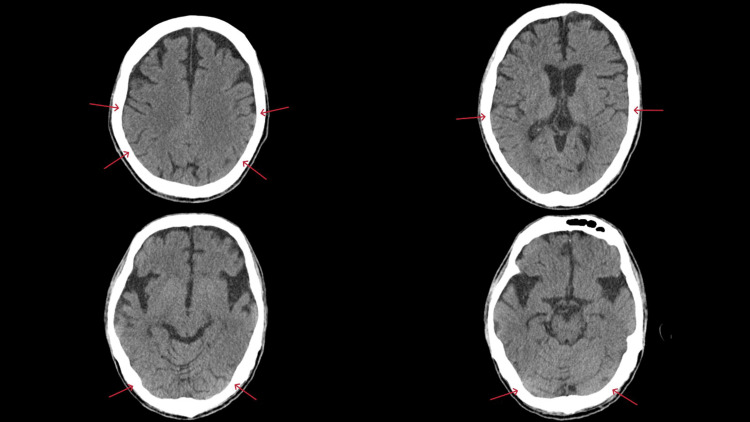
CT scan images after treatment show complete resolution of brain edema Compared to the CT scan taken at the time of arrival, the sulci are clearly visible (red arrows), suggesting improvement of brain edema.

His level of consciousness improved to GCS E4VTM6 on Day 1, and we extubated on same day. On Day 3, a repeat head CT showed complete resolution of brain edema (Figure 2), and he was discharged from the ICU on the same day. On Day 6, the patient was discharged back to his care facility without neurological sequelae.

## Discussion

This case highlights several important points. First, acute symptomatic hyponatremia can present with reversible brain edema on imaging. Second, brain edema in this context does not necessarily indicate a poor neurological prognosis. Finally, CT findings may aid in guiding management decisions in hyponatremia.

The differential diagnoses of hyponatremia are wide-ranging, and water intoxication, including psychogenic polydipsia, is a known cause of hyponatremia. Among psychiatric patients in care facilities, polydipsia prevalence has been reported at 15.7%, with 90.4% of affected individuals diagnosed with schizophrenia [1]. According to Rangan et al., 52% of adults with hyponatremia due to water intoxication had chronic psychiatric disorders, mainly schizophrenia spectrum disorders [2]. This patient, with schizophrenia and excessive water intake, fits this clinical pattern. Although syndrome of inappropriate antidiuresis (SIAD), hyperthyroidism, drug-induced hyponatremia, and Addison's disease could not be ruled out, we primarily considered water intoxication based on the patient's clear history of excessive water intake and prior episodes of water intoxication. In patients with water intoxication, urine osmolality is typically low (generally below 100 mOsm/kg), but acute physical stress factors such as convulsions can transiently increase urine osmolality due to vasopressin release [3]. In this case, although urine osmolality was not decreased on arrival, stress from the convulsions episode may have been a contributing factor.

Hyponatremia is defined as a serum sodium <135 mEq/L and is classified by severity: mild (130-135 mEq/L), moderate (125-129 mEq/L), and severe (<125 mEq/L). It is also categorized as acute or chronic, depending on the time until onset, with 48 hours often used as the cutoff point [4].

Clinical symptoms of hyponatremia range from mild to severe, with convulsions occurring more frequently as severity increases, and occur in up to 18% of cases with Na <109 mEq/L [5]. Although severe hyponatremia can cause brain edema, few cases have reported corresponding imaging findings. In this case, a head CT scan performed to evaluate impaired consciousness revealed typical diffuse brain edema. Based on the patient’s history of excessive water intake, we identified water intoxication as the likely cause of the brain edema. However, if the medical history had been unclear or unavailable, the findings may have been misinterpreted as resulting from convulsions, hypoxic encephalopathy, or meningitis. In addition, brain edema on imaging may have led to an underestimation of the patient’s neurological prognosis. Even in the absence of a clear medical history, water intoxication should be considered when diffuse brain edema is observed in patients with hyponatremia. Notably, despite the presence of brain edema in this case, the patient's general condition improved following admission and treatment. Thus, brain edema in the context of water intoxication does not necessarily indicate a poor neurological prognosis [6].

The recommended correction rate for hyponatremia is 10-12 mEq/L per 24 h in the US guidelines and 10 mEq/L in the European guidelines to prevent osmotic demyelination syndrome (ODS) [4,7]. However, some recent reports support more rapid correction in certain cases, making this topic controversial [8,9]. The European guidelines advise initiating treatment for severe or symptomatic hyponatremia with a bolus of 3% saline solution, along with frequent monitoring of sodium levels [4].

In this case, the patient presented with symptomatic severe hyponatremia and was initially treated with hypertonic saline (10% NaCl 60 mL + 500 mL normal saline, yielding approximately a 1.9% saline solution). As the sodium correction progressed more rapidly than expected, we transitioned to standard saline infusion and administered intranasal desmopressin.

In cases of water intoxication-induced hyponatremia, rapid serum sodium elevation is often observed due to polyuria, which can result in unintended overcorrection [10]. While hypertonic saline is generally recommended for severe or symptomatic hyponatremia, water restriction alone may be sufficient when water intoxication is the underlying cause [7]. In the emergency setting, head CT is commonly performed to evaluate impaired consciousness. If the brain edema is observed in conjunction with hyponatremia, and even if the patient's medical history is unclear, water intoxication should be considered as a potential cause. In such cases, CT findings may help guide the decision between initiating hypertonic saline or implementing water restriction as the primary treatment strategy.

When diffuse brain edema and convulsions are present in a patient with hyponatremia, interpreting CT findings can be challenging [11]. In the present case, both hyponatremia and convulsions likely contributed to the observed brain edema, suggesting a multifactorial etiology. Nonetheless, careful assessment of the patient’s history and urine output can assist in identifying water intoxication. In similar cases, close monitoring of urine volume and the rate of sodium correction over time is essential. If the patient exhibits polyuria and spontaneous correction appears likely, the use of hypertonic saline may be reconsidered.

## Conclusions

Diffuse brain edema can accompany acute hyponatremia, but its presence does not necessarily indicate a poor neurological prognosis. In patients with hyponatremia and imaging evidence of brain edema, water intoxication should be considered as a potential underlying cause.When hyponatremia is caused by water intoxication, treatment may not always require hypertonic saline; water restriction alone may suffice. Based on CT findings, a treatment strategy incorporating water restriction should be considered while monitoring serum sodium correction rate and urine output.
